# COVID-19 and Open Notes: A New Method to Enhance Patient Safety and Trust

**DOI:** 10.2196/29314

**Published:** 2021-06-21

**Authors:** Charlotte Blease, Liz Salmi, Maria Hägglund, Deborah Wachenheim, Catherine DesRoches

**Affiliations:** 1 Division of General Medicine Beth Israel Deaconess Medical Center Boston, MA United States; 2 Department of Women's and Children's Health Uppsala University Uppsala Sweden; 3 Harvard Medical School Boston, MA United States

**Keywords:** COVID-19, patient portals, electronic health records, patient safety, patient-centered care

## Abstract

From April 5, 2021, as part of the 21st Century Cures Act, all providers in the United States must offer patients access to the medical information housed in their electronic records. Via secure health portals, patients can log in to access lab and test results, lists of prescribed medications, referral appointments, and the narrative reports written by clinicians (so-called open notes). As US providers implement this practice innovation, we describe six promising ways in which patients' access to their notes might help address problems that either emerged with or were exacerbated by the COVID-19 pandemic.

COVID-19 has left an indelible legacy on patient care. Tragic numbers of lives have been lost to the virus, and it is not known how many excess deaths are due to reduced access to services or patients delaying health care [1]. With enforced isolation and savage stresses on essential workers, mental health and well-being have been affected [2]. The pandemic has also given rise to a new chronic condition: *long COVID*. Prevalence and susceptibility are poorly understood but patients may experience persistent symptoms including fatigue, body or muscle pains, insomnia, headaches, depression, and anxiety for months after their acute illness [3]. Compounding these problems are racial inequities in vaccine distribution and fears among certain segments of the public about the trustworthiness of vaccines [4,5]—a reticence, recent evidence suggests, is heightened among white politically conservative men [6].

Despite all the tragedy wrought by COVID-19, the pandemic has been a catalyst for innovating new practices in medicine.

In Spring 2020, in the space of a few short weeks, with the enforcement of physical distancing and stay-at-home rules to avoid transmission of the virus, patients and doctors were compelled to use different forms of communication [7]. In the United States, for example, telemedicine—the use of phones and videos for clinical visits—soared from fewer than 2% of primary care visits in 2019 to more than 35% by April 2020 [8]. Complementing telemedicine, and perhaps overshadowed by the pandemic, another practice innovation—allowing patients to access their own clinical records—is also taking hold [9]. As of April 5, 2021, as part of the 21st Century Cures Act, all providers in the United States must offer patients access to the medical information housed in their electronic records [10,11]. Via secure health portals, patients can log in to access lab and test results, lists of prescribed medications, referral and appointment times, and the words written by clinicians (so-called *open notes*). With few permitted exceptions, information that was previously hidden from view and the eyes of patients must now be available.

Open notes are not without precedent. By December 2020, around 55 million patients across 260 health systems in the United States and Canada were offered access to their clinical notes. In other countries, such as Sweden, the practice is advanced, and during the pandemic, studies show substantial increases in use of the nationwide health portal [12]. Prepandemic, approximately 80,000 new users logged into the Swedish portal each month; since June 2020, this number steadily reached more than 100,000 new users per month, peaking in November 2020 at 165,000. Health systems in the United States have seen similar upticks in patient portal registration throughout the pandemic, in some cases due to the technology for telemedicine visits being offered only through portal log-in. This indicates that the pandemic not only increased use among already existing portal users but also spurred adoption among new patients.

Promising evidence suggests that open notes might play a role in mitigating challenges with care, some of which emerged with the pandemic and some which have been exacerbated by COVID-19. We reflect on six ways in which open notes might help meet some of the challenges of COVID-19.

1. In light of the uptick in telemedicine, with social distancing measures in place, patients and clinicians have been compelled to rapidly navigate new modes of visits, and it is not yet known whether the quality of patient-clinician communication has been affected. Against these uncertainties, there is compelling evidence that open notes may help strengthen clinician-patient exchanges [9]. Large-scale online surveys conducted prior to the pandemic show that when patients can readily access their notes they better understand and remember their care plan, including the reasons why treatments have been prescribed and when (and why) referrals and follow-up appointments are scheduled [13,14].
2. During the pandemic, when many face-to-face ambulatory visits are still delayed or not possible, caregivers have faced considerable challenges in supporting family and friends. Unable to accompany patients to clinical visits or see loved ones in the hospital, they have lost important opportunities to find out what is going on and to fully participate in care. However, open notes may help to close this information gap. In studies, considerable numbers of patients report sharing notes with others [13]. Such access may be even more important for older adult patients or those who rely on care partners by helping them, and their carers, to understand treatment plans and to remember to access test results or follow up with referrals.
3. Open notes could also play a crucial role in closing the chasm of health disparities further deepened by COVID-19 [15]. Compared with White patients, Black, Hispanic, and Asian patients in the United States are more likely to report that reading their notes enhances trust in their doctors. In surveys, historically marginalized patients such as those who identify with an ethnic or racial minority group, persons with lower incomes, those with fewer years of formal education, or those whose first language differs from their clinician are the most enthusiastic about open notes when they log in to portals [16,17]. On the other hand, the “digital divide” in health care—driven by lack of broadband internet access [18], lack of email address or use, and low digital literacy—remains a barrier to the adoption of patient portals among these patient populations [19]. Although there is evidence this digital divide is narrowing, with greater commitment to safety net interventions such as patient outreach programs or the distribution of WiFi-enabled tablets, patients who have the most to gain from reading their notes could reap the benefits.
4. COVID-19 has taken a toll on mental health. However, patients with anxiety or depression who read their notes may benefit from better understanding their clinicians’ recommendations, helping them to stay on track with their care plan. In a large-scale survey of nearly 20,000 patients prescribed medications, 14% of patients who accessed their notes reported doing a better job adhering to their medications [20]. For patients with mental health diagnoses the figures were higher: 20% of patients with serious mental illnesses such as major depression, bipolar disorders, or schizophrenia and 18% of patients with other mental health diagnoses said they were more likely to take their prescribed medications after reading their clinical notes [14]. Preliminary findings from psychotherapy also show that open notes can improve the therapeutic alliance between patients and therapists, help build trust, and deepen patients’ understanding about what goes on in therapy sessions [21].
5. Patients experiencing symptoms of long COVID may feel marginalized or uncertain about their health care. In multiple surveys, patients who access open notes report feeling more empowered and engaged with managing their health [13,22,23]. As an asynchronous communication tool, open notes can serve as an online extension of the visit, helping improve a sense of control and engagement with care. This may be especially important for offering reassurance to patients with long COVID whose symptoms are still poorly understood [24,25].
6. Working under the considerable pressures wrought by the pandemic, it is not yet known whether patient safety has been negatively affected by human error [9]. Studies conducted prior to the pandemic show that around 1 in 5 patients who read their notes can, and do, perceive errors [26]. With more eyes on the charts, patients can help clinicians to correct inaccuracies or omissions and improve the feedback loop in care—all the more essential as a patient safety mechanism when routines have been disrupted and clinicians and patients are laboring under high levels of stress.

The effects of COVID-19 will be with us for years to come, but perhaps the most important memorial will be clinicians, patients, and care partners seizing on novel opportunities to improve care. Open notes do not offer a magic solution to the challenges of the pandemic, but by partnering with patients and families, through an invitation to read notes and be on the same page as their clinicians, it may offer an important new strategy to improve engagement, increase trust, and demonstrate respect for patients.
